# Web-Based Self-Management Guide for Kidney Transplant Recipients (The Getting on With Your Life With a Transplanted Kidney Study): Protocol for Development and Preliminary Testing

**DOI:** 10.2196/13420

**Published:** 2019-06-24

**Authors:** Daniela Massierer, Ruth Sapir-Pichhadze, Vanessa Bouchard, Kaberi Dasgupta, Nicolas Fernandez, Deborah da Costa, Sara Ahmed, Marie-Chantal Fortin, Rosalie Langevin, Nancy Mayo, Tania Janaudis-Ferreira

**Affiliations:** 1 School of Physical and Occupational Therapy McGill University Montreal, QC Canada; 2 Centre for Outcomes Research and Evaluation Research Institute of the McGill University Health Centre Montreal, QC Canada; 3 Canadian Donation and Transplantation Research Program Edmonton, AB Canada; 4 Division of Nephrology and Multi-Organ Transplant Program Department of Medicine McGill University Montreal, QC Canada; 5 Hôpital de Chicoutimi Centre intégré universitaire de santé et services sociaux du Saguenay-Lac-Saint-Jean Chicoutimi, QC Canada; 6 Division of Endocrinology & Metabolism McGill University Health Centre Montreal, QC Canada; 7 Division of Internal Medicine McGill University Health Centre Montreal, QC Canada; 8 Metabolic Disorders and Complications Research Institute of the McGill University Health Centre Montreal, QC Canada; 9 Department of Family Medicine and Emergency Medicine University of Montreal Montreal, QC Canada; 10 Department of Medicine University of Montreal Montreal, QC Canada; 11 Division of Clinical Epidemiology McGill University Health Centre Montreal, QC Canada; 12 Division of Geriatrics McGill University Health Centre Montreal, QC Canada; 13 Translational Research in Respiratory Diseases Program Research Institute of the McGill University Health Centre Montreal, QC Canada

**Keywords:** self-management, kidney transplantation, eHealth, quality of life, exercise, physical activity

## Abstract

**Background:**

Although it is well known that compared with dialysis, kidney transplantation improves the quality of life (QoL) of patients with end-stage renal disease, posttransplant recovery of physical health and other aspects of QoL remain well below age- and sex-matched norms. In addition, most transplant recipients are not physically active even years after the transplant and face several barriers to engaging in physical activity (PA). This is of concern as low levels of PA in transplant recipients has been associated with increased risk of mortality and poor graft function. Optimization of QoL needs a team approach involving the patients and the members of the health care team. While members of the health care team are focused on optimizing the biological responses to transplant, patients may have few or no tools at their disposal to engage in behaviors that optimize QoL. To accomplish the need of supporting these patients in the self-management of their condition and to facilitate engagement with PA, new tools tailored to this population are required.

**Objective:**

The aim of this protocol study is to develop a Web-based, patient-centered self-management intervention to promote a healthy lifestyle, increase daily PA, and improve QoL in kidney transplant recipients.

**Methods:**

We will use the Obesity-Related Behavioral Intervention Trials model for developing behavioral treatments for chronic diseases to guide the proposed project. We will follow a modified version of the iterative 10-step process that was used to develop educational material for people with multiple sclerosis. The development of the intervention will occur in partnership with patients and a multidisciplinary team of clinicians and researchers. A comprehensive needs assessment including data from our pilot study, literature review, and focus groups will be conducted. The focus groups will be conducted with 6 to 10 participants for each type of stakeholders: patients and professional experts to identify areas of concerns of kidney transplant recipients that are appropriate to address through self-management. The areas of concern identified through the assessment needs will be included in the website.

**Results:**

This study has received funding from the Kidney Foundation of Canada for 2 years (2018-2020) and was recently granted ethics approval. Investigators have begun conducting the needs assessment described in step 1 of the study. The study is expected to be completed by the end of 2020.

**Conclusions:**

This will be the first comprehensive, evidence- and experience-based self-management program for kidney transplant recipients. Once the intervention is developed, we anticipate improvements in patient experience, shared decision making, daily PA, QoL, and, in future studies, improvements in health outcomes and demonstrations of cost savings in posttransplant care.

**International Registered Report Identifier (IRRID):**

PRR1-10.2196/13420

## Introduction

### Benefits of Kidney Transplantation to the Individual and Society

In 2016, there were a total of 2835 solid organ transplants performed in Canada [1]. Of those, more than 61.05% (1731/2835) were kidney transplants. Kidney transplantation doubles life expectancy of the recipient compared with dialysis and, importantly, leads to considerable cost savings [1].

### Shift From Transplant Outcomes and Mortality to Quality of Life

Although kidney transplantation undoubtedly prolongs life of people with end-stage renal disease (ESRD) and confers great economic benefits to the society [1], the challenges experienced by recipients do not end after the transplantation. With considerable advances in organ preservation, surgical techniques, and immunosuppressive therapy, short-term survival following solid organ transplant has greatly improved [2]. As a result of improved graft survival and reduced deaths from infection or rejection, health care professionals and researchers have started to shift their focus toward reducing morbidity from cardiovascular disease and sustaining improvements in quality of life (QoL) [3-5].

### Quality of Life After Kidney Transplantation

Compared with dialysis, transplantation improves the QoL of patients with ESRD; however, physical health and other aspects of QoL among transplant recipients remain below age- and sex-matched norms [5-8]. Our group recently used a personalized measure of QoL, the Patient Generated Index (PGI) questionnaire (Multimedia Appendix 1), to evaluate 51 kidney transplant recipients (mean age 58.6, SD 11.6 years; 1 to 3+ years after transplant). We aimed to identify particular areas of QoL that are affected by having experienced a kidney transplant and found that 71% (36/51) of the kidney transplant recipients reported at least one health concern. The top identified areas were physiological complaints (eg, urine infection and abnormal blood tests), nutrition, mobility, fatigue, pain, and mood/emotions [9].

### Decreased Levels of Physical Activity

Low physical activity (PA) levels in transplant candidates and recipients are associated with important clinical outcomes including increased wait-list mortality [10,11], cardiovascular and all-cause mortality after transplant [4], and poor posttransplant outcomes [12]. Living a physically active lifestyle can help mitigate secondary chronic conditions that arise after transplant, such as high blood pressure, glucose intolerance/diabetes, osteoporosis, sarcopenia and fatigue, [13] as well as improve aerobic capacity and QoL in transplant recipients [13]. Despite numerous health benefits, most transplant recipients are not physically active even years after transplant and face several barriers to engaging in PA [14]. Our group showed, in a survey of 113 Canadian solid organ transplant recipients (65 men and 47 women) who were aged between 40 and 70 years and 1 to 5 years posttransplant, that 60% of participants were engaging in low levels of PA, and 16% had activity levels lower than what has been observed in frail individuals [14]. In addition, a large number of these individuals had never engaged in light to strenuous exercise or strengthening exercises [14].

### Barriers to Being Physically Active and Poor Availability of Rehabilitation Programs

Helping people adopt a physically active lifestyle requires an understanding of the barriers to and facilitators of PA. Sullivan and colleagues [15] have described that in people with chronic health conditions, environmental obstacles (not knowing where to exercise), time constraints (believing there is not enough time to exercise), and social limitations (not having support for exercise) are important barriers that need to be addressed in tailored PA interventions. We have found that transplant recipients report cost (of exercise facilities), side effects of medications, and lack of knowledge/guidelines on exercise after transplant as the major barriers to engaging in PA [14]. In the same study, “motivation to stay healthy” and “physician recommendation” were cited as the leading facilitators to being more physically active [14]. However, in a follow-up survey with Canadian transplant physicians [16], we found that while most of the transplant physicians reported that they counsel transplant recipients about PA, only 27% provided counseling to 100% of their patients, and 18% felt confident in doing so. Lack of time and lack of specific exercise guidelines to transplant recipients were identified as the main barriers to PA counseling. In addition, based on a Canadian survey, there are very few dedicated solid organ transplant rehabilitation programs in Canada [17]; most programs are for heart and lung transplant recipients, with only 1 program available for liver recipients and none for kidney recipients. Therefore, based on the barriers and gaps identified at the patient-health care professional—and health care system—levels, kidney transplant recipients require support in developing skills to be able to enhance their lifestyle. To accomplish the need of supporting these patients in self-managing their health burdens and to engage with PA, new easily accessible tools are required.

### Self-Management—An Ideal Intervention to Improve Quality of Life in Kidney Recipients

Given the comprehensive nature of the problems experienced by kidney recipients and that every aspect of QoL can be negatively affected, a self-management intervention is the natural approach to improve QoL in these individuals [18]. Self-management is a lifelong task where the individual takes responsibility to manage the symptoms, treatment, physical and psychosocial consequences, and lifestyle changes inherent in living with a chronic condition. It focuses on the development of core skills related to self-assessment, goal setting, problem solving, decision making, resource utilization, partnership between patient and health care provider, and action plans [19]. The benefit of self-management for kidney transplant recipients is that it is individualized and focuses on enhancing the ability of individuals to improve their health status, regardless of where the individual is on the journey after transplant. Although self-management has been shown to be effective in many chronic conditions, a comprehensive, evidence- and experience-based self-management program that includes support to increase levels of PA is not available for kidney transplant recipients anywhere in the world. We found only 2 papers in the literature describing the development of a self-management program for kidney transplant recipients. Schmid-Mohler and colleagues [20] developed a self-management program in Switzerland for kidney recipients who were in their first year after transplant; however, this program is only available in German, has a limited focus on the prevention of weight gain, increasing PA, and medication adherence and does not offer an electronic health app for symptom monitoring. A study from the Netherlands [21] described a self-management online support system, but this study also had a limited approach. In this study, kidney recipients were asked to use a blood pressure monitor and a creatinine measurement device at home according to a fixed schedule. In Canada, the scenario is not different. The self-management tools that are available in Canadian Transplant Centers where kidney transplant is performed are mainly focused on medication management, side effects identification, medication interaction awareness, and infection risk (oral or email communication with collaborators in each site). Although very important, these topics do not cover other areas of concern that are important to patients such as mobility, PA, fatigue, pain, and mood/emotions. This demonstrates a gap in care after transplant in Canada and the need for new tools to assist kidney transplant recipients build the skills and attitudes required to effectively self-manage different aspects of their lives after the transplant.

The overarching goal of our study is to develop a patient-centered intervention to increase daily PA and improve QoL in kidney transplant recipients. We propose to develop Getting on with your life with a transplanted kidney (GETONTRAK), a Web-based program for the promotion of PA and self-management in kidney transplant recipients. The specific aims are (1) to develop a comprehensive Web-based guide for the promotion of PA and self-management in kidney transplant recipients and (2) to examine the changes in daily step counts and QoL after delivering the GETONTRAK self-management program to kidney transplant recipients to collect preliminary data for a future pilot randomized controlled trial.

## Methods

### Overview

The study will take place at the Research Institute of the McGill University Health Centre (RI-MUHC) in Montreal, Canada, between December 2018 and December 2020. The study received ethics approval by the MUHC Research Ethics Board (2019-4875).

**Figure 1:**
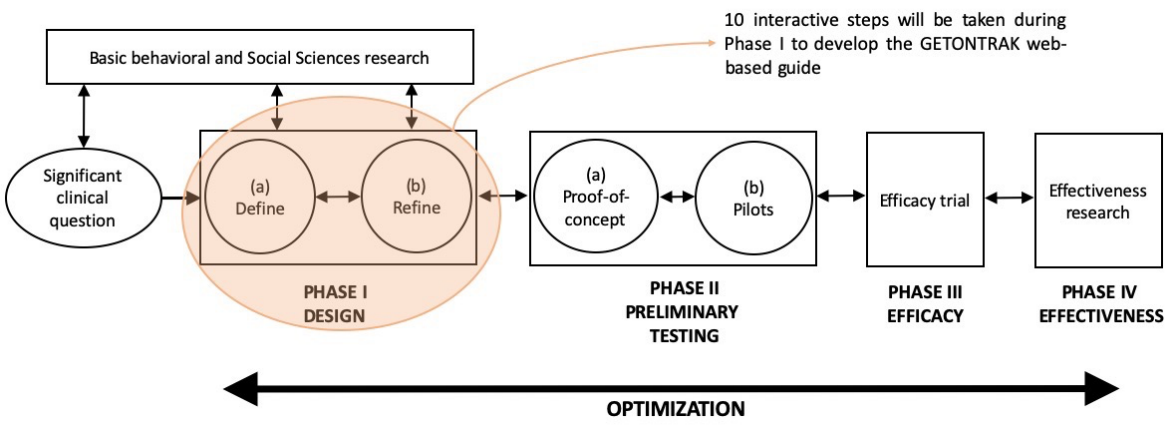
The Obesity-Related Behavioral Intervention Trials model. GETONTRAK: Getting on with your life with a transplanted kidney.

We will use the Obesity-Related Behavioral Intervention Trials (ORBIT) model for developing behavioral treatments for chronic diseases [22] to guide the proposed project and develop our Web-based guide, the GETONTRAK. The ORBIT model provides guidance on the process of treatment development using a progressive and transdisciplinary approach. Our project falls into phase I of the ORBIT model, which is the design phase of a behavioral intervention (Figure 1).

To meet the goal of phase I (design and define the intervention), we will follow a modified version of the iterative 10-step process that was used to develop educational material prepared for people with multiple sclerosis [23]. These steps will include (1) assessment of the needs of the population, (2) format development, (3) obtaining content for the topics, (4) content adaptation to fit the purpose, (5) obtaining feedback, (6) finalizing the content for the topics, (7) website screen design, (8) translation to French and peer review evaluation, (9) preliminary testing, and (10) integrating feedback to update the website.

### The 10-Step Process

#### Step 1: Assessing the Needs of the Population

We will use several methods to identify the needs of kidney transplant recipients that are appropriate to address through self-management. As a starting point, we will conduct a systematic literature search of the literature on the physical and psychological impairments that kidney recipients experience after transplantation (during first year or later), affected areas of QoL, and side effects of medications. We will also use data from our pilot study that aimed to identify the particular areas of QoL that are affected by the experience of a kidney transplant. Our pilot study uncovered a large number of unmet needs (kidney recipients identified physiological issues, nutrition, mobility, pain, and mood/emotions as being the most common affected areas of their lives after the kidney transplant [9]. These data will be used to help create the topics to be addressed in the self-management guide. In addition, we will conduct focus groups with patients, clinicians, and researchers to confirm the topics identified through the review and pilot work and/or identify new ones. Our plan is to conduct between 4 and 6 focus groups for each type of stakeholders (patients and clinicians/researchers), which will last about 1.5 hours. All focus groups will be conducted at the MUHC, and informed consent for participation will be obtained at the MUHC. The focus groups will consist of 6 to 10 participants and 1 or 2 moderators. The moderators will work to create a climate of mutual respect and facilitate the discussion among the participants. Data gathering will be considered complete when (1) data saturation is reached (ie, new focus groups do not provide additional information) and (2) there is sufficient internal diversification in terms of respondent characteristics (sex, age, profession, etc). Our sampling strategy will be purposive. For example, we will select experienced professionals (at least 2 years working with kidney transplant patients) with different backgrounds (eg physicians, pharmacists, physiotherapists, occupational therapists, psychologists, nurses, and social worker), sex, age, and from different regions in Canada. We will also aim to recruit kidney transplant recipients (of both sexes, different age and ethnic background and different time after transplant) through the Patient Partnership Platform of the Canadian Donation and Transplantation Research Program (CDTRP), MUHC and Centre Hospitalier de l'Université de Montréal (CHUM), and the Canadian Network for Rehabilitation and Exercise for Solid Organ Transplant Optimal Recovery (CAN-RESTORE) website and social media. To ensure consistency in the focus group meetings, the same facilitator will run all the focus group meetings using a meeting script (Multimedia Appendix 2). The focus group sessions will mostly be in English and will be audio-recorded, and 2 additional researchers will participate to take notes to capture nonverbal data. Participants will be excluded if they are unable or have limited ability to speak English. Due to the nature of focus groups, it is impossible to guarantee complete confidentiality as other members of the group will be aware of your identity. However, all participants are instructed to keep what is said in the focus group confidential.

##### Qualitative Data Analysis

Data collection and analysis will occur concurrently and iteratively. This process will be done to allow the research team to identify new areas of discussion and determine when saturation has been achieved [24]. Detailed notes and the audio recordings will be analyzed using the content and thematic analysis method described by Miles and Huberman [25]. Employing this method, the investigators will (1) establish a list of themes that will constitute the coding frame; (2) read the notes, listen to the audio recordings, and sort them according to this coding frame to create a more abstract frame of analysis; (3) add new themes or categories as they emerge from the notes; (4) organize these categories into figures, charts, or matrices; and (5) draw corresponding conclusions. A list of codes will be developed according to the research questions. To meet the quality criteria (validity and reliability) for qualitative research, we will present our results to different groups of stakeholders [24,26]. Following this comprehensive process of step 1, we will create a list of topics for inclusion in the Web-based self-management guide. These topics may be relevant to any stage after transplant (within first year of transplant or later). In addition to the topics related to the recipients’ concerns after transplant, we will include generic topics relevant to self-management to enhance kidney recipients’ capacity to take charge of their health (including promotion of PA). These topics will address self-management skills and attitudes of self-assessment, goal setting, motivation, and developing an action plan.

#### Step 2: Develop the Format

We will work with Expression Web Solutions, a specialized company in offering digital solutions, and plan the format of the website. A preliminary website architecture is shown in Multimedia Appendix 3. The website will include 5 key sections that will cover developing self-management skills as well as the specific topics chosen in step 1. The sections are described below:

##### Getting Started

This first section will introduce self-management and be designed to orient the new user to the goals and skills needed for self-management. Patients will identify areas of QoL (using a personalized measure of QoL, the PGI) they think are affected to identify the content of the guide that are most relevant to them. Thereafter, they will be directed to an inventory of facilitators and barriers that influences their approach to life after transplantation, form goals, and build an action plan.

##### Healthy Life Style for All

This section will include topics that are relevant to anyone with or without a chronic disease such as healthy eating and drinking, sleep, stress reduction, and challenging your mind and body.

##### Dealing With Posttransplant Challenges

This section will include content to help deal with the challenges that the life after transplant may bring to kidney transplant recipients. The topics of this section will be identified during step 1. It will contain information about the specific topic (“Did You Know?”) and the research evidence (“Research Evidence Shows”). It will also include a self-assessment tool (“How Am I Doing?”) and information on what to do (“What Can I Do?”) and how to do it (“How Can I Do it?”).

##### Partnering Effectively With Your Health Care Team

This section will help transplant recipients develop the necessary skills to become an active member of the health care team and will include topics such as how to get the most out of each health care meeting, managing uncertainty, understanding the vocabulary, and tests doctors use and learning how to use online resources.

##### Putting It All Together

This last section will show the user how to build their personal plan of action by prioritizing goals, monitoring progress, and making adjustments as needed.

#### Step 3: Obtain Content for the Topics

In addition to the investigative team, clinicians and researchers with expertise in kidney transplantation will be identified through our center (MUHC), the CHUM, CDTRP, and published literature. Content will be informed by published systematic reviews. When systematic reviews are not available, experts will conduct their own review to guide the content for each topic. These experts are not considered to be research participants but rather expert consultants, and they will be paid accordingly.

#### Step 4: Adapt the Content to Fit the Purpose

The content provided by the experts will be edited by the coprincipal investigators and other members of the investigative team and rewritten in layperson’s language, aiming at a grade 6 or 7 reading level. Medical terms will be defined. Link or references to other good electronic resources and webpages will be included into the page rather than repeating the information.

#### Step 5: Obtain Feedback

A minimum of 2 patient partners and 1 caregiver will be recruited from the Patient Partnership Platform of the CDTRP and the CAN-RESTORE website to provide feedback on the content of each topic. Specifically, they will provide feedback on the relevance of the content as well as on the usefulness, clarity, and applicability of the information. This feedback will be used to make changes to the content. In case of major reorganization or rewriting, a second round of patient feedback will be performed. Participants will be informed that they are participating in a study and that agreeing to provide feedback will serve as their consent to participate in the study.

#### Step 6: Finalize the Content for the Topics

At this stage, the coprincipal investigators and other members of the investigative team will review the feedback provided by the patient partners and caregivers and incorporate them into the content for each topic. Suggestions for images and formatting will be made in preparation for screen design.

#### Step 7: Screen Design

When the content of each topic is finalized, it will be sent to Expression Web Solutions for screen design. The website prototype will be reviewed by the research team and 2 patient partners for ease of reading, image choice, and placement and usability.

#### Step 8: Translation to French and Peer Review Evaluation

Once the website prototype is finalized, it will be translated to French. We will contact national and international experts in clinical content and self-management who will not have been involved with the development content of the website to evaluate the extent to which the GETONTRAK Web-based guide is consistent with the evidence; up to date, useful, and clear with respect to their messages and actions; and with a low risk of harm. These experts will fill out a Web-based survey to obtain structured feedback based on the Patient Education Materials Assessment Tool (PEMAT) [27]. The PEMAT uses a systematic approach to evaluate the understandability and actionability of patient education materials. Shoemaker and colleagues [27] suggested a cutoff of 70% for understandability and actionability. The reviewers will be identified through the published literature and during the focus groups with professionals (they will be asked to list names of potential reviewers). A minimum of 2 reviewers will be assigned per topic for each version of the website (English and French). Monetary compensation will be offered to enhance participation.

#### Step 9: Preliminary Testing

We will recruit a convenience sample of 10 kidney transplant recipients (eg, 5 French and 5 English speakers) from the outpatient clinic of the MUHC to use the GETONTRAK website as intended and provide feedback on their experience as users. GETONTRAK website will be open access to all its components. To ensure that selected participants are representative of the outpatient clinic, attention will be paid to selecting participants of different sexes, ages, levels of PA, time after transplant, and sociocultural backgrounds. We will select patients with and without computer/internet literacy. A brief explanation of the GETONTRAK online guide will be given to patients before the testing period. Informed consent for participation will be obtained at the MUHC outpatient clinic.

For baseline assessment (T0), in addition to collecting demographic data, patients will complete 3 QoL questionnaires (PGI, EuroQol five-dimensional questionnaire, and Kidney Transplant Questionnaire) and will be asked to wear a PiezoRxD Pedometer on the waist for 7 days consecutive and at least for 12 hours per day. At this time, patients will not have seen the pedometer-based walking program offered in the GETONTRAK Web-based guide. After the initial assessment is completed, patients will meet with the research coordinator, be asked about their self-management habits, and receive instructions on how to use the website, including the pedometer-based walking program. They will be asked to use the GETONTRAK website for 2 months. After 2 months (T2), patients will complete the 3 QoL questionnaires again, and daily steps data from the last week of the 2-month period will be used for comparison with the baseline data. To monitor adherence to the pedometer-based walking program, patients will be asked to complete a diary with information on days and times they could not wear the PiezoRxD Pedometer and respective reasons. At T2, patients will be asked about the acceptability and usability of the Web-based self-management guide. We will use a mixed-methods approach comprising self-report questionnaire, semistructured telephone interview (Multimedia Appendix 4), and system usage data to assess acceptability and usability. The online survey administered at T2 will assess usability (layout, navigation, functionality, and features) and acceptability (overall usefulness, usefulness of specific topics, utility of the site for improving PA and other aspects of their life, credibility, and program length). Responses to each item will be rated on a Likert scale ranging from 1 (strongly disagree) to 5 (strongly agree). The telephone interview will inquire about the most helpful sections, barriers and reasons for nonuse, or discontinued use and solicit suggestions for additions, deletions, and improvements. The online survey and interview questions are guided by previous usability/acceptability studies [28,29] and recommendations from the Science Panel on Interactive Communications and Health [30] for website usability evaluation. Self-reported questionnaires and the interviews will be available in English and French.

#### Step 10: Integrate Feedback to Update the Website

At this stage, we will incorporate the feedback received, transmit the modification to the health technology partner, and a new version of the website will be produced. The investigators intend to update the content of the website periodically.

## Results

This study has received funding from the Kidney Foundation of Canada for 2 years (2018-2020) and recently been granted ethics approval. Investigators have begun to conduct the needs assessment described in step 1 of the study (January-April 2019). The study is expected to be completed by the end of 2020.

The GETONTRAK Web-based guide will improve information delivery, monitoring of symptom progression, and patient empowerment. By using our educational material, kidney recipients will develop 5 core skills: problem solving, decision making, resource utilization, forming patient/health care provider partnership, and taking action. Our knowledge tool has great potential to improve health outcomes and patient experience after transplantation as well as to reduce health care costs as patients will take charge of their health and may have fewer medical visits. However, these outcomes will be evaluated in future studies.

## Discussion

### Impact

The steps taken in this project will ensure that a comprehensive, evidence- and experience- based self-management program is available for kidney transplant recipients in Canada and other parts of the world.

### Potential Challenges

The coinvestigators, NM and VB, who developed the process with 10 iterative steps for the development of global self-management programs [23], shared their lessons learned, which are as follows: (1) involve patient partners early on to ensure relevance of the topics, (2) have a structure and a template to plan and organize the content, (3) get a Web-design team involved early on so the format is developed along with the content, (4) there is no need to abandon medical language; rather it should be explained and simplified. (5) Recruitment is always a challenge in any clinical study, so to minimize recruitment issues and ensure consistent participation, offer an honorarium for patient partners, study participants, experts (content writers), and peer-reviewers. Finally, the 10 steps are iterative and allow for several opportunities for feedback and consequently improvements in the content and format of the website.

Once the GETONTRAK is developed and its benefits confirmed (future randomized control trial [RCT]), the authors will need to focus on the dissemination and implementation of the guide to all transplant centers and clinics in Canada and around the world to ensure that health care professionals and patients are aware of this new resource. After the self-management is developed and implemented in clinical practice, some potential challenges may appear. Some individuals may have limited access to technological resources or may show lower general engagement with health care and health-related interventions. Strategies to mitigate barriers to technological access should be taken into consideration when offering the GETONTRAK intervention. These strategies may include offering a workbook or rented computer/tablets instead. Other barriers such as side effects of medication may interfere with the willingness of patients to participate in interventions that involve PA [14] as they may not feel well enough to become more active. Nevertheless, patients will have access to many other topics in the GETONTRAK self-management guide, which will give them the opportunity to improve their QoL regardless of their readiness to be part of a PA program.

### Next Steps

When step 10 is finalized, the investigators will move to phase II of the ORBIT model (Figure 1) and conduct an RCT to pilot-test the GETONTRAK website. This RCT is not part of this protocol and will include outcomes such as adherence, patient empowerment, QoL, PA, and health care utilization. As research is always evolving, the investigators intend to update the content of the website periodically.

## Appendix

Multimedia Appendix 1 Patient Generated Index (PGI) questionnaire.

Multimedia Appendix 2 Description of the script of the focus group meetings.

Multimedia Appendix 3 Preliminary website architecture.

Multimedia Appendix 4 Semistructured telephone interview.

Multimedia Appendix 5 Peer-reviewer report from the Kidney Foundation of Canada.

## Abbreviations

CDTRP: Canadian Donation and Transplantation Research Program
CHUM: Centre Hospitalier de l'Université de Montréal
ESRD: end-stage renal disease
GETONTRAK: Getting on with your life with a transplanted kidney
MUHC: McGill University Health Centre
ORBIT: Obesity-Related Behavioral Intervention Trials
PA: physical activity
PEMAT: Patient Education Materials Assessment Tool
PGI: Patient Generated Index
QoL: quality of life
RCT: randomized controlled trial
