# Supplementary figures and images for: Web-Based Self-Management Guide for Kidney Transplant Recipients (The Getting on With Your Life With a Transplanted Kidney Study): Protocol for Development and Preliminary Testing
Source: JMIR Res Protoc. 2019 Jun 24;8(6):e13420. doi: 10.2196/13420 (PMC6613326; doi:10.2196/13420)

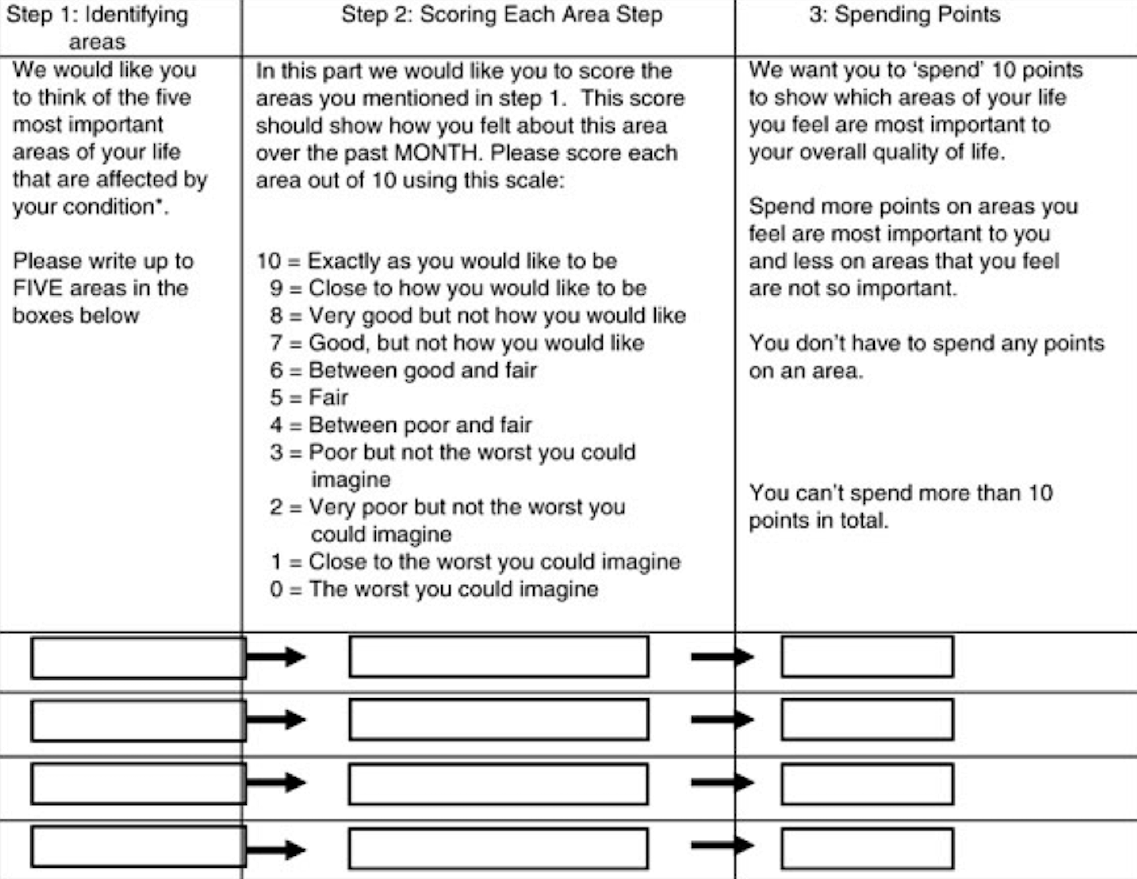

Supplement: Multimedia Appendix 1 [file resprot_v8i6e13420_app1.png]

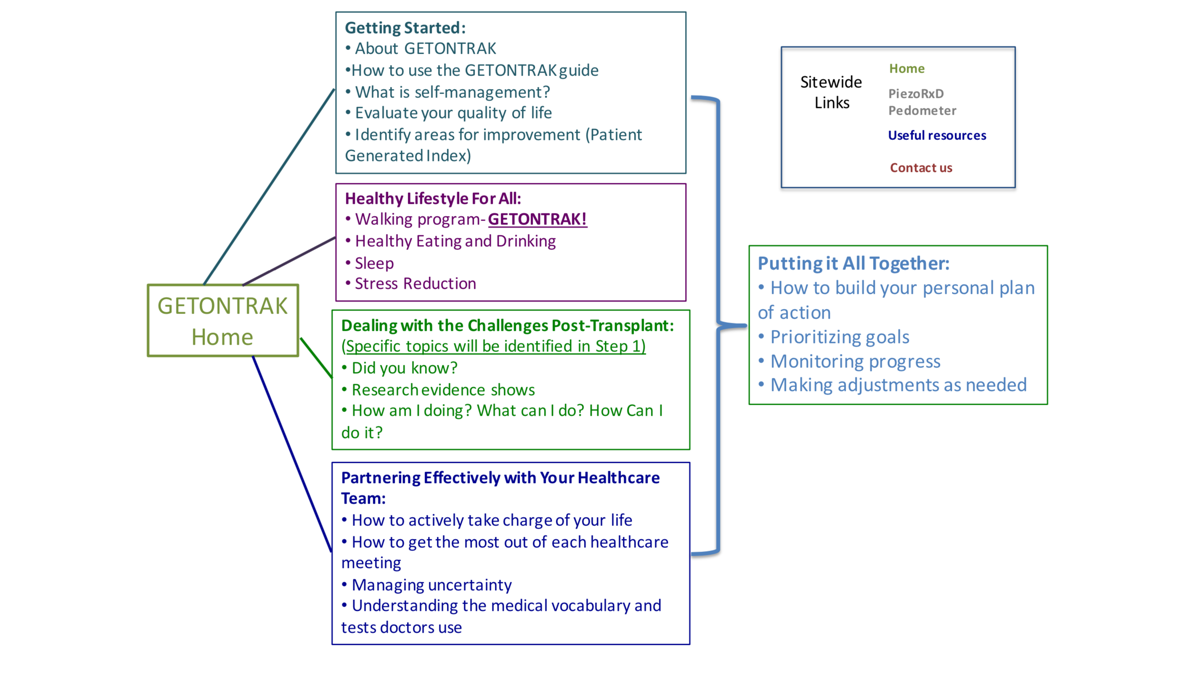

Supplement: Multimedia Appendix 3 [file resprot_v8i6e13420_app3.png]
