# Supplementary material for: Web-Based Self-Management Guide for Kidney Transplant Recipients (The Getting on With Your Life With a Transplanted Kidney Study): Protocol for Development and Preliminary Testing
Source: JMIR Res Protoc. 2019 Jun 24;8(6):e13420. doi: 10.2196/13420 (PMC6613326; doi:10.2196/13420)
Supplement: Multimedia Appendix 2 [file resprot_v8i6e13420_app2.pdf]

Appendix 2. Focus group meeting script (Step 1).

Focus Group Outline
DRAFT

*The focus groups will occur separately for the two "types" of stakeholders: 1) patients and 2) professionals (clinicians and researchers).

Draft Outline:

- Moderators introduction
- Introduction to the GETONTRAK project
- Purpose of the focus group
- Ground rules (discussion should flow freely, confidentiality, practical aspects of the meeting (bathrooms, time, etc).
- Introduction of participants
- Moderators present the findings of the systematic reviews (part of Phase 1) and pilot work and list potential topics that could be included in the GETONTRAK self-management guide.
- Examples of focus group questions:

  Focus Group with patients:

  - ✓ Please talk about your recovery post-kidney transplant
  - ✓ Did you have an easy or difficult recovery?
  - ✓ Which aspects of your life were affected after the transplantation?
  - ✓ Did you receive support from the healthcare team to deal with the problems you faced after transplantation? Which problems you wished you had received support for, but you did not?
  - ✓ About the list of topics that the moderators presented: Do you think the topics are relevant and should be included in the GETONTRAK self-management guide? Are there any topics that you think should NOT be included? Why? Are there topics that were not listed but should be included? If so, which ones?

  Focus Group with professionals (clinicians/researchers)

  - ✓ Please talk about the challenges that your patients/clients experience after transplantation.
  - ✓ What kind of problems do they complain about when they see you at your clinic?
  - ✓ Do you feel you have the right tools to help your patients face these challenges?
  - ✓ About the list of topics that the moderators presented: Do you think the topics are relevant and should be included in the GETONTRAK self-management guide? Are there any topics that you think should NOT be included? Why? Are there topics that were not listed but should be included? If so, which ones?
- Closing
