# Supplementary material for: Web-Based Self-Management Guide for Kidney Transplant Recipients (The Getting on With Your Life With a Transplanted Kidney Study): Protocol for Development and Preliminary Testing
Source: JMIR Res Protoc. 2019 Jun 24;8(6):e13420. doi: 10.2196/13420 (PMC6613326; doi:10.2196/13420)
Supplement: Multimedia Appendix 4 [file resprot_v8i6e13420_app4.pdf]

Appendix 4. Self-report online questionnaire and semi-structured telephone interview drafts.

Acceptability and Usability Self-reported Online Questionnaire (DRAFT)

In the last 2 months we have provided you with online access to ***GETONTRAK*** and we would now like to ask you some questions about the website. We would like you to access the website now so you may refer to it while you are completing this form.

All information provided in this form will be kept strictly confidential. This survey will take approximately 20-30 minutes.

Thinking about the GETONTRAK we would like you to rate on a scale of **1 (strongly disagree) to 5 (strongly agree)** the following.

	**Strongly Disagree**				**Strongly Agree**
1. *GETONTRAK* website is easy to use.	1	2	3	4	5
2. I did not need technical support to use it.	1	2	3	4	5
3. People will learn to use *GETONTRAK* very quickly.	1	2	3	4	5
4. The various functions in *GETONTRAK* are well integrated.	1	2	3	4	5
5. The information provided in the *Getting Started* section helped me decide which section to focus on.	1	2	3	4	5
6. The information provided in the *Healthy Lifestyle for All* is credible.	1	2	3	4	5
7. The information provided in the *Healthy Lifestyle for All* is relevant to kidney transplant recipients	1	2	3	4	5
8. The information provided in the *Dealing with the Challenges Post-Transplant* is credible.	1	2	3	4	5
9. The information provided in the *Dealing with the Challenges Post-Transplant* is relevant to kidney transplant recipients	1	2	3	4	5
10. I learned useful tools to improve my health from the *GETONTRAK* website	1	2	3	4	5
11. *GETONTRAK* improved my knowledge about healthy behaviors (i.e. sleep, physical activity)	1	2	3	4	5
12. The information on the *GETONTRAK* website will help me maintain better health habits.	1	2	3	4	5
13. I liked the Walking Program	1	2	3	4	5
14. Walking Program helped me get or stay more active	1	2	3	4	5

15. The information provided in the *Partnering Effectively with Your Healthcare Team* section helped me understand how I can actively take charge of my health	1 2 3 4 5
16. The information provided in the *Putting it together section* helped me build and prioritize my personal goals	1 2 3 4 5
17. The information provided in the *Putting it together* section helped me monitor my symptoms	1 2 3 4 5
18. The information provided in the *Putting it together* section helped me monitor my daily steps	1 2 3 4 5
19. The *GETONTRAK app* helped me monitor my symptoms	1 2 3 4 5
20. The *GETONTRAK app* helped me monitor my daily steps	1 2 3 4 5
21. The *GETONTRAK* website helped me to know what questions to ask my healthcare provider about my health concerns	1 2 3 4 5
22. The *GETONTRAK* website helped me to know what questions to ask my healthcare provider about my healthy lifestyle behaviours	1 2 3 4 5
23. I was satisfied with the amount of information provided on *GETONTRAK*	1 2 3 4 5
24. I would recommend *GETONTRAK* to other kidney transplant recipients who are seeking reliable information on optimizing their health post-transplant	1 2 3 4 5

Share with us some of your ideas on how we can improve GETONTRAK.

____

____

____

THANK YOU FOR YOUR PARTICIPATION. YOUR RESPONSES ARE EXTREMELY IMPORTANT TO THE SUCCESSFUL DEVELOPMENT OF *GETONTRAK*

Acceptability and Usability <u>Telephone Interview</u> (DRAFT)

Thank you for completing the online questionnaire about the GETONTRAK website. In the last 2 months we have provided you with on-line access to *GETONTRAK* and we would now like to ask you some questions about the website.

We would like you to access the website now so you may refer to it during this interview.

All information provided in this interview will be kept strictly confidential. This interview will take approximately 20 minutes.

1. Is there anything you think is missing from the GETONTRAK website?

2. Were there any sections where you felt there was too much or too little information or advice?

3. What parts did you find most helpful to you and why?

4. What changes would you recommend?

5. Is there anything else you would like to say about the website that we did not cover in the previous questions?

THANK YOU FOR YOUR PARTICIPATION. YOUR RESPONSES ARE EXTREMELY IMPORTANT TO THE SUCCESSFUL DEVELOPMENT OF *GETONTRAK.*
