# Supplementary material for: Web-Based Self-Management Guide for Kidney Transplant Recipients (The Getting on With Your Life With a Transplanted Kidney Study): Protocol for Development and Preliminary Testing
Source: JMIR Res Protoc. 2019 Jun 24;8(6):e13420. doi: 10.2196/13420 (PMC6613326; doi:10.2196/13420)
Supplement: Multimedia Appendix 5 [file resprot_v8i6e13420_app5.pdf]

REFEREE REPORT ON A GRANT APPLICATION / RAPPORT D'ÉVALUATEUR SUR UNE DEMANDE DE SUBVENTION

Name of candidate / Nom du candidat : Janaudis-Ferreira, Tania

The objective of the proposed research is to develop a knowledge tool (a workbook) for building the skills and attitudes required for kidney transplant recipients to effectively self-manage, with the goal of improving quality of life. It builds on pilot data from 51 kidney transplant recipients (publication under development). The project brings together a well-balanced and senior team to support the new investigator. The application is exceptionally well-written and easy to follow. There are no fatal flaws, but could use minor clarification in a few places.

STATEMENT OF OBJECTIVES AND SIGNIFICANCE OF CONTRIBUTION

- Both the overarching goal and aim are clearly stated
- Significance of the work is clearly outlined, supported with the literature, and aligned with patient priorities (CNTRP 2014) and KFOC

BACKGROUND INFORMATION AND LITERATURE REVIEW

- Background is concise and tailored to show need for the proposed work. Very persuasively written.
- Quality of life is clearly defined

METHODOLOGY

- The Orbit model clearly depicts the plan to enact workbook development
- Step 1: the team identifies that they will begin with a systematic review of impairments kidney recipients experience after transplantation. What kind of systematic review will they undertake? Depending on this methodology and synthesis, it could take a lot longer than 4 months as outlined in the timeline
- Sample size for Step 9, preliminary testing of workbook with kidney recipients, is not identified

ANALYSIS

- Given that a workbook is to be developed, an analytic plan is not the main focus. However, there were a few places where analysis was not addressed. For example: Both for focus groups in Step 1 and interviews in Step 9, how will qualitative data be analyzed? For example, with content or thematic analysis? (Ethical approval is clearly needed for these steps, yet not mentioned.) Further, an overarching qualitative methodology could provide guidance with this regard.
- Sex and gender considerations, mitigation strategies, and KT plan are all exceptional

QUALITY OF APPLICANT AND INSTITUTIONAL SUPPORT

- Very strong, multi-disciplinary team
- I have not seen collaborator letters before where people are committing to attend a focus group?
- The letter from CNTRP is particularly strong with commitment to resources (ie website, teleconference system, access to patient-researchers in CNTRP), as well as collaboration with Dr. Bouchard who created a similar program in MS.
- While the proposal relates to development of a workbook, the collaborator letters refer to a workbook and development of an e-health app. I suspect the e-health app was removed from this KFOC application, but kept in for their CIHR submission, and not removed from attached letters of collaboration?

- Dr. Fernandez, kidney recipient, is an excellent addition to the research team, and his perspective will help enact patient-oriented research principles

BUDGET

- While a post-doctoral fellow is referenced in the timeline, this is not identified in the budget
- There is very little RA support here for the planned work, and the work seems under-resourced (i.e. 7 hours/week year 1, 14 hours/week year 2)
- Rather than paying $1080 to bring in 4 clinicians (? – not sure who) to participate in focus groups, consider putting these resources towards the RA and conduct a focus group with these individuals via ZOOM or skype with video. These sessions can also be recorded.

Allied Health Research Grant Competition 2018

Allied Health Scientific Committee Meeting

Title: *Getting on with your life with a transplanted kidney: GETONTRAK. Development of a self-mangement workbook to improve quality of life in kidney*

Applicant: Janaudis-Ferreira, Tania

COMMENTS

A really well written, strong grant, significance clearly articulated, very persuasive, alignment with KFOC clearly stated. Sex and gender considerations, knowledge translation, mitigations are all clearly outlined. Some confusion regarding an E-health app was noted. Additional patient participation in the development of the focus groups was suggested. Minor clarifications and suggestions regarding the budget are included in the written reviews.

REFEREE REPORT ON A GRANT APPLICATION /
RAPPORT D'ÉVALUATEUR SUR UNE DEMANDE DE SUBVENTION

Name of candidate / Nom du candidat :

Tania Janaudis-Ferreira

- Mixed of collaborators from different specialties and different centres/ very multidisciplinary. However, I questioned how involved will some of the collaborators be. From their support letter, some of them seem to only be participating to a focus group and not be involved in the development/input for the research project.

-Good research questions in line with the KFOC and CNTRP objectives
-Building on work that this group has completed on QOL post-transplant, they are trying to find resolution in some of the issues identified in a previous study.

- I would encourage for more patient involvement in this study and for patient collaborator to be involved earlier in the process, especially since the objectives of this study are so patients focus.
